# Functional speech disorder in a patient with X-linked adrenomyeloneuropathy: a diagnostic challenge

**DOI:** 10.1016/j.prdoa.2026.100479

**Published:** 2026-07-03

**Authors:** Juan Sebastián Sánchez León, Ingrid Lorena da Silva Gomes, Artur Francisco Schumacher Schuh, Daniel Teixeira-dos-Santos, Lucia Tesolin, Maira Rozenfeld Olchik

**Affiliations:** a Graduate Program in Medical Sciences, Federal University of Rio Grande do Sul, Porto Alegre, Brazil; b Department of Neurology, Hospital de Clínicas de Porto Alegre (HCPA), Porto Alegre, Brazil; c Department of Neurology, Functional Neurological Disorders Clinic, General Hospital of Bolzano, Bolzano, Italy

## Abstract

We describe a 54-year-old female carrier of X-linked adrenomyeloneuropathy presenting with episodic stuttering triggered by mild exertion. Despite pre-existing neurological disease, normal neuroimaging, clinical inconsistency, distractibility, and rapid response to respiratory-based therapy supported a functional speech disorder. This case emphasizes the importance of recognizing functional speech disorders in patients with other comorbid neurological conditions.

X-linked adrenomyeloneuropathy (X-AMN) is a rare peroxisomal disorder caused by pathogenic variants in the *ABCD1* gene, affecting the metabolism of very long-chain fatty acids (VLCFA). While male patients often present with severe cerebral involvement in childhood or adrenal insufficiency X-linked adrenoleukodystrophy (X-ALD), heterozygous female carriers may develop milder, slowly progressive neurological symptoms later in life, typically manifesting as spastic paraparesis, gait disturbances, or bladder dysfunction related to X-AMN [1]. Functional neurological disorders (FND) have an estimated prevalence of 50 / 100,000, and their incidence is approximated at 4–12 / 100,000 cases. Women can represent up to 75% of the cases [2]. Functional speech disorders (FSD) are a subtype characterized by abnormalities in speech production that cannot be explained by conventional macroscopic structural lesions of the nervous system. However, it may be associated with alterations in functional connectivity across neural networks and/or structural brain morphology, and can be diagnosed through positive clinical signs demonstrating inconsistency with recognized neurological diseases [3], [4]. Regrettably, FND/FSD are often under-recognized, particularly in patients with concurrent neurological diagnoses.

We report the clinical case of a 54-year-old woman with a past medical history of asthma, a previous episode of deep vein thrombosis (DVT), and three separate episodes of pulmonary embolism. Since the age of 18, the patient had been experiencing a slowly progressive course of lower-limb spasticity, gait disturbance, and a general decline in motor function. This prompted further investigation, and after other etiologies were ruled out, the patient was diagnosed with X-AMN, with genetic confirmation obtained at the age of 32. She subsequently began follow-up with the genetics and neurology services, during which her symptoms continued to progress, and a gradual decline in motor function was observed over the years.

At the age of 49, she developed speech disturbances characterized by mild exertion-triggered episodes of stuttering. It was observed that she presented with unremarkable speech; however, after minimal physical effort, such as motor tasks during the evaluation, the patient exhibited lip tremor and limb postures resembling dystonia in both hands and feet, followed by transient stuttering. This stuttering was characterized by syllable repetitions, variability in speech fluency, and brief blocks between some syllables or words **(Video 1)**. These episodes showed marked variability during the examination, and improved with distractibility maneuvers. Notably, prominent facial grimacing was also associated with these events. Her oxygen saturation and heart rate remained stable during the episodes, and no other physiological changes were noticed. Writing and reading abilities remained preserved. Activities such as light physical exercise or even the effort required during strength testing in the neurological examination were sufficient to precipitate these episodes.

The patient had several predisposing factors, including the uncertainty surrounding her own illness and her son's condition (her son has the cerebral form of X-linked adrenoleukodystrophy and is currently 15 years old), as well as a history of depressive symptoms that required psychological support. Neurological examination remained largely unchanged from previous assessments, demonstrating pyramidal signs, including lower limb spasticity, generalized hyperreflexia, and bilateral Hoffman and Babinski signs. Brain MRI showed no cerebral abnormalities associated with X-linked cerebral adrenoleukodystrophy and no findings that could explain the speech disturbances, prompting a thorough investigation of other conditions, including neuromuscular disorders, pneumophonatory incoordination, laryngeal dystonia, and others. The patient underwent extensive assessment by various medical professionals over time, with no conclusive diagnosis, leading to the decision for a multidisciplinary evaluation with speech and language therapists. This case report was approved by the local Ethics Committee (protocol 2025-0442), and the patient provided written informed consent for publication of the case details and video material.

After several evaluations, and once a non-functional neurological disorder was ruled out, the diagnosis of functional speech disorder was established based on the positive clinical features identified during the examination. She was advised to use breath-control mobile applications and to engage in breathing exercises and meditation practices to manage the speech disturbance **(Video 1).** Following these interventions, she demonstrated significant improvement in speech. She continues to follow up with speech therapy, focusing on respiratory training during spontaneous speech as well as maintaining multidisciplinary follow-up.

Speech disturbances are uncommon in X-ALD and X-AMN and, when present, are often attributed to structural neurological progression [1]. In this case, a 54-year-old X-AMN female developed episodic, stress-induced stuttering consistent with FSD, despite pre-existing neurological deficits secondary to her condition. The speech disturbances coincided with subtle limb postures, normal neuroimaging findings, stable physiological parameters during episodes, and rapid improvement following respiratory and mindfulness-based interventions, supporting the diagnosis of a functional speech disorder.

In many cases, establishing the diagnosis of a FSD can be challenging, as several diseases or conditions may cause or mimic stuttering [4], [5]. It is therefore essential to consider these differential diagnoses. In our case, the presence of an underlying neurological disease initially led to the assumption that her symptoms were attributable to the primary condition or a complication of it, which likely contributed to a delay in reaching the correct diagnosis.

FSD can manifest as dysfluencies, dysphonia, or prosodic abnormalities, despite preserved respiratory and phonatory function. Positive diagnostic features include abrupt onset, attention-dependent variability, and responsiveness to non-pharmacological treatments; the present case aligns with these characteristics [4], [5].

Current evidence suggests that respiratory muscle incoordination plays a significant role in stuttering phenomena, supporting the involvement of a motor-functional mechanism supporting a possible treatment avenue focused on respiratory control. Past reports on functional voice and speech disorders highlight the efficacy of behavioral voice therapy combined with top-down therapeutic approaches, such as respiratory training and psychotherapy [5]. Our patient's improvement following breathing and meditation exercises further corroborates these findings.

Although female carriers of X-AMN may develop late-onset spastic paraparesis and mild ataxia, speech disturbances are rare; thus, this case highlights the need to consider functional speech disorders when new-onset speech abnormalities occur in patients with established neurological disease, particularly in the presence of clinical inconsistency and therapeutic reversibility [2], [4], [5].

Differentiating between non-functional and functional speech disorders requires attentive clinical evaluation, identification of positive signs of functional etiology, and early interdisciplinary intervention. Management focusing on respiratory control, mindfulness, and cognitive education can yield rapid and meaningful improvement.

The following are the supplementary data related to this article.

Supplementary video S1

Supplementary material 1

Supplementary material 2

Supplementary data to this article can be found online at https://doi.org/10.1016/j.prdoa.2026.100479.

## CRediT authorship contribution statement

**Juan Sebastián Sánchez León:** Writing – original draft, Formal analysis, Data curation, Conceptualization. **Ingrid Lorena da Silva Gomes:** Writing – review & editing, Visualization, Validation. **Artur Francisco Schumacher Schuh:** Validation, Investigation. **Daniel Teixeira-dos-Santos:** Writing – review & editing, Validation. **Lucia Tesolin:** Writing – review & editing, Validation. **Maira Rozenfeld Olchik:** Writing – review & editing, Validation, Methodology, Investigation, Conceptualization.

## Funding

This study received institutional support from the Hospital de Clínicas de Porto Alegre (ROR: https://ror.org/010we4y38). No specific external funding was received for this work.

## Declaration of competing interest

The authors declare that they have no known competing financial interests or personal relationships that could have appeared to influence the work reported in this paper.
